# Multimodal physiotherapy versus scapular and conventional exercise for persistent pain following breast cancer treatment: a randomized controlled trial

**DOI:** 10.1007/s11764-026-02035-z

**Published:** 2026-05-23

**Authors:** Virginia Prieto-Gómez, María José Yuste-Sánchez, Laura Lorenzo-Gallego, Fernando Ramos-Gómez, Beatriz Navarro-Brazález, Deborah Falla, María Torres-Lacomba

**Affiliations:** 1 Physiotherapy in Women’s Health Research Group, Physiotherapy Department, University of Alcalá, Alcalá de Henares, Madrid Spain; 2 Ramón y Cajal Institute of Health Research – IRYCIS, Ramón y Cajal University Hospital, Madrid, Spain; 3 Department of Physiotherapy, Medicine and Biomedical Sciences, Faculty of Physiotherapy, University of A Coruna, A Coruña, Spain; 4 Centre of Precision Rehabilitation for Spinal Pain (CPR Spine), School of Sport, Exercise and Rehabilitation Sciences, College of Life and Environmental Sciences, University of Birmingham, Birmingham, UK

**Keywords:** Breast cancer survivors, Persistent pain, Multimodal physiotherapy, Scapular exercise, Dry needling, Neurodynamic techniques, Pain neuroscience education

## Abstract

**Objective:**

To evaluate the effectiveness of a multimodal physiotherapy program compared with exercise programs on pain, disability, quality of life, and shoulder muscle activation in women with persistent pain following breast cancer treatment (PPBCT).

**Design:**

Randomized, assessor-blinded, three-arm parallel-group clinical trial.

**Setting:**

Physiotherapy in Women’s Health Research Unit at the University of Alcalá, Madrid, Spain.

**Subjects:**

Ninety women with PPBCT were recruited between April 2016 and April 2022.

**Interventions:**

Participants were randomized into three groups (*n* = 30 each). All groups received six individual physiotherapy sessions over 6 weeks and the same pain education. Participants were allocated to (i) multimodal physiotherapy (MP) including scapular exercises, deep dry needling, and neurodynamic techniques, (ii) scapular exercises (SE), or (iii) conventional shoulder exercises (CSE).

**Outcome measures:**

The primary outcome was shoulder pain intensity (VAS). Secondary outcomes included perceived shoulder pain and disability assessed with the Shoulder Pain and Disability Index (SPADI), health-related quality of life measured with the Functional Assessment of Cancer Therapy-Breast version 4 (FACT-Bv4), shoulder muscle activation assessed using surface electromyography, and myofascial pain syndrome evaluated according to Travell and Simons’ criteria.

**Results:**

Ninety women were randomized and completed all follow-up assessments. All three groups showed improvements over time in most outcomes. Overall, the pattern of results favored MP, followed by SE and then CSE, with consistent superiority of MP across most outcomes and several time points. For the primary outcome, MP showed greater reductions in pain intensity than SE at all assessment points, with large between-group effects post-intervention and at 3 months, and a reduced effect at 6 months. Both MP and SE showed greater pain reductions than CSE at all assessment points, with large between-group effects, and changes exceeded the minimal clinically important difference only in the MP and SE groups.

**Conclusions:**

A multimodal physiotherapy program combining scapular exercises, deep dry needling, and neurodynamic techniques was more effective than exercise programs alone in women with PPBCT. Scapular exercise-based rehabilitation appears to play a key role in long-term recovery, while the addition of other physiotherapy techniques may primarily contribute to earlier symptom reduction. These findings support the use of a multimodal approach to address the complex mechanisms underlying persistent pain in breast cancer survivors, with potential implications for clinical decision-making in survivorship care.

**Implications for cancer survivors:**

A multimodal physiotherapy approach integrating scapular exercises, pain education, deep dry needling, and neurodynamic techniques may provide clinically meaningful benefits for breast cancer survivors with persistent pain, improving function, quality of life, and long-term engagement in self-management strategies.

**Supplementary Information:**

The online version contains supplementary material available at https://doi.org/10.1007/s11764-026-02035-z.

## Introduction

Chronic pain is a frequent consequence following breast cancer treatment with a reported prevalence ranging from 25 to 60% [1]. This condition is commonly referred to as *Persistent Pain following Breast Cancer Treatment* (PPBCT) and is characterized by pain and sensory disturbances affecting the breast, chest wall, axilla, or arm on the affected side. PPBCT frequently includes neuropathic pain resulting from neural tissue damage during surgery [2]. However, surgical procedures and adjuvant therapies may also induce additional tissue injury, contributing to the development of nociceptive and nociplastic pain mechanisms [3, 4].

PPBCT is typically managed through pharmacological treatment; however, outcomes are often unsatisfactory [5], likely due to the diverse underlying pain mechanisms. Tailoring treatment to the predominant pain mechanisms may therefore be a key factor in improving outcomes. An individualized approach, taking into account the various mechanisms—neuropathic, nociceptive, or nociplastic—would enable the selection of more appropriate interventions and could contribute to improvements in pain, function, and health-related quality of life [6]. This approach is particularly relevant in the management of PPBCT, where pain mechanisms can vary significantly between women [4].

In this context, physiotherapy has gained recognition as an alternative treatment strategy, with therapeutic exercise being widely recommended for shoulder pain unrelated to oncological treatment [7]. However, based on the literature, experimental studies on upper limb pain following breast cancer surgery have predominantly focused on surgery-related sequelae, mainly pain, reduction in range of motion, and loss of function, without specifically addressing the profile of persistent post-treatment pain characteristic of the population under study. Additionally, studies that include upper limb exercises have largely concentrated on conventional exercises for the glenohumeral joint, aimed at improving strength, endurance, and flexibility, with marked heterogeneity in prescription principles [8].

Available evidence on the inclusion of scapular exercises for persistent shoulder pain in non-oncological populations suggests that therapeutic scapular exercise may offer advantages over conventional exercises in reducing pain and improving shoulder function [9, 10]. This advantage may be related to the importance of the scapulothoracic joint in overall shoulder function, given its ability to restore scapular positioning, recover synchronous movement during arm elevation, optimize local motor control, and restore the length-tension relationship of the periscapular muscles [9, 10]. A recent meta-analysis by Zhong et al. showed moderate evidence that scapular stabilization exercises can improve shoulder pain and disability compared to conventional or non-specific scapula approaches in subacromial pain syndrome [9]. Similarly, a systematic review with meta-analysis by Melo et al. suggested that scapular therapeutic exercises may benefit shoulder function compared to other interventions [10]. Given the relevance of the scapula in shoulder function [9, 10] and the scapular dysfunction present in women with PPBCT [11], it is plausible to hypothesize that including scapular exercises may offer additional benefits, both in combination with other treatments and as a standalone approach.

In addition to exercise therapy, other physiotherapy techniques may address mechanisms involved in persistent pain following breast cancer treatment. Emerging studies indicate that myofascial pain syndrome (MPS) may also occur in this population [1, 12] together with increased mechanosensitivity of neural tissue [13–15] which may contribute to the persistence and exacerbation of symptoms [3, 15]. Dry needling has been proposed as an adjunct intervention for the treatment of MPS. Although its effectiveness has been supported in several musculoskeletal conditions [16], no consistent evidence has been found regarding its use in managing PPBCT. Furthermore, neurodynamic techniques aim to reduce neural mechanosensitivity and modulate pain [17–19], but their potential role in the management of PPBCT remains unclear. Combining these approaches with therapeutic exercise may therefore enhance treatment outcomes by addressing multiple pain mechanisms.

The biopsychosocial model provides an essential perspective for understanding the complexity of persistent pain in breast cancer survivors. This model recognizes that pain is not solely the result of biological factors but is also influenced by psychological and social factors. Anxiety, depression, pain catastrophizing, and fear of movement can significantly affect both pain perception and the effectiveness of interventions. Additionally, social factors such as social support, work-related stress, and reintegration into pre-diagnosis roles can influence the experience and persistence of pain [5].

In this regard, pain education has become a fundamental component in the management of persistent pain and is widely recommended in current clinical guidelines. Pain neuroscience education, integrated within the biopsychosocial approach, is crucial not only for understanding the origin of pain but also for improving coping mechanisms, increasing perceived self-efficacy, reducing fear of movement, and fostering treatment adherence [5, 19, 20]. De Groef et al. emphasize that pain education should be part of the standard of care for breast cancer survivors with persistent pain, given its positive impact on both pain prevention and management [5]. Similarly, Balordi et al. suggest that education could help women better understand their pain and improve their coping abilities without adverse effects [19].

However, to date, no randomized clinical trials have compared a multimodal physiotherapy approach with different exercise-based interventions in women with PPBCT.

Taking all of the above into consideration, the aim of this study was to evaluate the effectiveness of a multimodal physiotherapy intervention—including scapular-focused exercises, deep dry needling, neurodynamic techniques, and pain education—compared with scapular-focused exercises plus pain education and conventional shoulder exercises plus pain education. All interventions were based on a common foundation of pain education, which current recommendations suggest as the standard of care. Additionally, a qualitative component was included to explore women’s experiences in maintaining home-based exercises and to identify factors influencing adherence.

## Methods

### Design

We conducted a single-center, randomized, assessor-blinded, three-armed parallel-group clinical trial of women with persistent pain following breast cancer treatment at the Physiotherapy in Women’s Health Research Unit at the University of Alcalá, Madrid, Spain, between April 2016 and April 2022. The study was conducted according to the Declaration of Helsinki and is reported according to the CONSORT guidelines.

Trial registration: ClinicalTrials.gov, NCT02735668, registered 14 April 2016.

### Participants

Participants were recruited at Príncipe de Asturias University Hospital, Alcalá de Henares, Madrid, Spain, through the Department of Gynecology and Obstetrics. Eligibility screening and confirmation of the inclusion and exclusion criteria were performed by a physiotherapist (PT1) who was not involved in the delivery of the interventions or outcome assessment. Eligible women with PPBCT (treated by either surgery and/or radiotherapy and/or chemotherapy) experiencing pain for at least 6 months were invited to participate in the study. In addition, women receiving endocrine therapy during the study were also included. The established exclusion criteria were as follows: shoulder pain prior to breast cancer treatment, bilateral breast cancer, predominant nociplastic pain identified according to the clinical classification guidelines for predominant neuropathic, nociceptive, or nociplastic pain described by Leysen et al. [21], active axillary web syndrome at the time of study inclusion [22], active lymphedema requiring treatment [23], any contraindications for conservative or invasive physiotherapy (infection, fever, metastasis, locoregional recurrence, wounds in the area of the puncture, metal allergy, or fear of needles), neurological disorders, current prescribed or regular intake of analgesic or anti-inflammatory drugs, that could alter the response of the nervous system, shoulder abduction range of motion less than 90°, and cognitive impairment preventing understanding of the study procedures or providing informed consent.

### Sample size estimation

With a sample size of 30 participants per group, we estimated 80% power to detect a between-group difference of 2.21 cm in the change of pain intensity measured with a Visual Analog Scale (VAS), and 90% power to detect a difference of 2.55 cm between treatments, assuming a standard deviation of the change of 3 cm. These values were defined a priori as plausible between-group differences in pain intensity, adopting a conservative approach in the absence of directly comparable previous trials. The calculation was performed using a standard *t*-test with a significance level of *α* = 0.05. The sample size calculation was performed using R version 4.2.0 (R Foundation for Statistical Computing, Vienna, Austria).

### Randomization and blinding

Following written informed consent, an equal number of women were randomly allocated using a computer**-**generated randomization sequence (EPIDAT version 3.1 Xunta de Galicia, Spain, 2010) to one of the three groups: (1) multimodal physiotherapy, (2) scapular exercises, or (3) conventional shoulder exercises. Randomization was also conducted by PT1.

### Assessment and data collection

Outcome measurement time points: Each group received a 6-week intervention consisting of one physiotherapy session per week (six sessions in total). Outcome measures were evaluated at baseline (A0), immediately after the intervention (A1), and at follow-up assessments 3 months (A2) and 6 months (A3) after the intervention (A1). A second physiotherapist (PT2) performed all outcome assessments and remained blinded to group allocation. Participants were instructed not to disclose their treatment allocation to the assessor.

#### Primary outcome

Participants reported their current *pain intensity* using a 10-cm VAS consisting of a 10 cm horizontal with the descriptors “no pain” on the left and “worst imaginable pain” on the right. The validity and reliability of the VAS have been widely reported. We considered a minimal clinically important difference (MCID) of 1.4 cm, based on studies in patients with rotator cuff-related shoulder pain [24].

#### Secondary outcomes

The *Shoulder Pain and Disability Index* (SPADI) is a self-administered questionnaire used to measure pain and disability associated with shoulder disorders. The SPADI contains 13 items that measure two domains: a 5-item subscale that assesses pain and an 8-item subscale that measures disability. SPADI is reliable, valid, and responsive for assessing shoulder pain and disability in Spanish women following breast cancer treatment [25]. A MCID of 17 points has been reported for patients with shoulder pain and disability [26].

*Health-related quality of life* was measured with the Functional Assessment of Cancer Therapy-Breast (FACT-B) Spanish version 4. FACT-Bv4 is a 44-item questionnaire designed to measure multidimensional HRQoL in women with breast cancer. Higher scores indicate better quality of life. FACT-Bv4 demonstrates ease of administration, brevity, reliability, validity, and sensitivity to change [27]. A MCID of 7–8 points has been reported in women with breast cancer [28].

In addition to the patient-reported outcome measures, the presence of MPS (active MTrP) was assessed in shoulder and axio-scapular muscles according to the criteria of Simons et al. [16], guided by each participant’s clinical pain distribution. Moreover, surface electromyography (sEMG) was used to measure the amplitude of the upper trapezius, lower trapezius, serratus anterior, infraspinatus and middle deltoid muscles during arm elevation performed in the scapular plane [29]. Bipolar Ag/AgCl electrodes (KendallTM 100 Series Foram Electrodes, Covidien, MA, USA) were used and positioned according to the recommendations of Cram and Kasman [30]. EMG data were acquired with a five-channel data recording system PowerLab 15 T (ADinstrument, Oxford, UK) at a sampling frequency of 2000 Hz [29] and processed using LabChart 7 software. The EMG signal was amplified with a gain of 1000, filtered (10–500 Hz), rectified, and smoothed, and the root mean square (RMS) was calculated [29]. A baseline recording was performed with the participant at rest (90° of arm elevation in the scapular plane). Participants then performed three repetitions of a 5-s isometric contraction at 90° of arm elevation in the scapular plane, with 5-s of rest between trials. The amplitude of activation was defined as the average RMS expressed as a percentage relative to baseline activity (RMS%).

#### Additional variables

At the baseline assessment (A0), demographic and identification data were collected including age, body mass index, and breast cancer therapies. Additional assessments included evaluation of scapular dyskinesis, static scapular position, scapular motor control, pain characteristics, and neural tissue mechanosensitivity.

#### Scapular dyskinesis

Scapular dyskinesis was assessed by evaluating the position of the scapula during shoulder flexion and abduction. Participants were classified as either pattern 1 (tilting), pattern 2 (winging), pattern 3 (dysrhythmia), pattern 4 (normal scapulohumeral rhythm), or pattern 5 (mixed pattern) [31].

#### Position of the scapula at rest

The position of the scapula was observed with the participant standing to determine possible alterations in the static orientation of the scapula. The scapula was considered to have excessive anterior tilt when the lower angle of the scapula was prominent dorsally [32], excessive internal rotation when the medial border of the scapula was prominent [32], excessive elevation when the superior angle of the scapula and/or the lateral edge of the acromion were located superiorly relative to the spinous process of the second thoracic vertebra [33], insufficient upward rotation when the medial border of the scapula was not parallel to the line of spinous processes of that same level or with a slight upward rotation (approximately 5°) [33], and excessive abduction when the distance between the root of the scapular spine and the spinous process of the corresponding vertebra exceeded 7.5 cm (Lennie test) [34].

#### Kinetic medial rotation test

This test was performed to assess control of the scapular muscles. The participant was positioned supine with the shoulder abducted to 90° and the elbow flexed to 90°. The participant’s shoulder was placed in 60° of internal rotation, and the participant was asked to maintain this position while keeping the scapula in a neutral position. The test was considered positive when the scapular moved into anterior tilt, downward rotation, or elevation [32].

#### Pain characteristics

Pain characteristics were recorded and classified according to the Guidelines for the Clinical Classification of Predominant Neuropathic or Nociceptive Pain described by Leysen et al. [21].

#### Neural tissue mechanosensitivity

Neural tissue mechanosensitivity was assessed using the Upper Limb Neural Tension Test (ULNT1). The test was performed with the participant in the supine position, following the standardized sequence: step 1: scapular stabilization; step 2: shoulder abduction (90°); step 3: wrist and finger extension; step 4: forearm supination; step 5: shoulder external rotation of the shoulder (70/90°); step 6: elbow extension. A neural sensitization maneuver was then performed by adding contralateral cervical side-bending. To be considered a positive neurodynamic test, symptoms had to be reproduced or exacerbated and further increased by the sensitization maneuver. If symptoms did not increase during the sensitization maneuver, a confirmatory neural desensitization maneuver consisting of ipsilateral neck side-bending was performed. If symptoms decreased during the desensitization maneuver, the test was considered positive, indicating that symptoms could be attributed, at least in part, to neural tissue involvement [35].

### Intervention

All interventions were performed by a physiotherapist (PT3) with more than 10 years of experience in these techniques. This physiotherapist was the only study member aware of group allocation. All groups received standardized patient education consisting of six individual one-to-one sessions of 30 min each. The content included the following: (1) basic knowledge about pain neurophysiology, movement impairments, cancer-related fatigue, and lymphedema related to oncological treatments; (2) individualized pain mitigation and management strategies according to each participant’s contributing and aggravating factors; (3) individualized strategies to support exercise continuation, including the use of an exercise diary, self-management strategies, self-monitoring, and recognition of signs and symptoms; and (4) healthy lifestyle habits [36].

### Multimodal physiotherapy program

#### Scapular exercises

Based on the evaluation of scapular dyskinesis and the kinetic medial rotation test, as well as the individual characteristics of each participant, individualized scapular exercises were prescribed using a motor control approach [32, 37, 38] with emphasis on achieving a neutral scapular orientation [32] (Supplementary data A).

#### Deep dry needling

Based on the assessment of active MTrPs, deep dry needling was performed according to previously described sterile procedures including standard aseptic technique, using sterile disposable needles [16]. Active MTrPs were identified following the criteria of Simons et al., including a palpable taut band, a hypersensitive tender spot, reproduction of the patient’s familiar pain, and referred pain consistent with the clinical presentation [16]. Muscle selection was guided by each participant’s pain distribution.

A deep dry needling technique was applied using sterile disposable needles. The needle was inserted into the MTrP, and multiple rapid insertions were performed to elicit local twitch responses, typically obtaining 4–5 responses per point when tolerated by the participant. Immediately after needle withdrawal, hemostatic compression and passive stretching were applied to the treated muscle.

The number of treated points per session was individualized according to clinical findings. Dry needling was applied once per session when indicated, within the six-session intervention period. Safety criteria were strictly followed, including prior screening for contraindications such as active lymphedema requiring treatment, infection, metastasis, or local tissue compromise. The procedure was performed by an experienced physiotherapist, and participants were continuously monitored to ensure that the intervention remained within a tolerable and safe range.

#### Neurodynamic techniques

When the ULNT1 was positive, neurodynamic techniques were included, which consisted of longitudinal nerve gliding techniques based on the ULNT1. With the participant in the supine position, the following standardized sequence was performed: step 1: scapular stabilization; step 2: shoulder abduction (90°); step 3: wrist and fingers extension; step 4: forearm supination; step 5: shoulder external rotation (70/90°); and step 6: elbow extension. Mobilization was by alternately decreasing contralateral cervical side-bending while increasing shoulder abduction, and vice versa, for approximately 90 s. The physiotherapist continuously monitored the participant to ensure that the technique remained pain-free. If pain occurred, shoulder abduction was reduced to remain within a non-painful range [39]. Sessions in this group lasted approximately 55 to 60 min.

### Scapular exercises

This group performed individualized scapular exercises following the same criteria described for the multimodal physiotherapy group and as outlined in Supplementary data A [32, 37, 38]. Session duration was approximately 35 to 40 min.

### Conventional shoulder exercises

Flexibility and strengthening exercises of the shoulder muscles, commonly used in the management of shoulder pain disorders, were performed, including shoulder joint mobilization exercises, isometric exercises, and resisted exercises using elastic bands (Supplementary data B) [40]. The sessions in this group also lasted approximately 35 to 40 min.

### Post-treatment exercise and adherence

After completing the supervised intervention, participants in all groups were encouraged to continue performing their prescribed exercises three times per week until the 6-month follow-up. Exercise adherence was monitored using an exercise diary completed by the participants. During this follow-up period, participants were able to contact the research team if they had any doubts or issues related to the program.

To better understand the factors that could facilitate or hinder adherence to home exercises, a qualitative study was embedded within the clinical trial. A descriptive qualitative design was used, combining focus groups and individual interviews [41]. The study followed the Standards for Reporting Qualitative Research (SRQR) guidelines [42] and the COREQ checklist [43].

### Qualitative interviews

Semi-structured individual and group interviews were conducted from a phenomenological perspective to explore participants’ experiences of performing home-based exercises after the intervention.

Individual interviews were conducted first, followed by group interviews with different participants, in order to obtain additional perspectives and richer data [44]. From May 2020 to November 2020, participants who attended the A3 assessment (6-month follow-up) were invited to participate.

Prior to the start of the interviews, an interview guide was developed by a group of three physiotherapists specialized in women’s health, a medical doctor specialized in pain management, and a gynecologist specialized in breast cancer treatment. PT4 conducted individual interviews, while PT5, a physiotherapist specialized in women’s health who was not involved in the clinical trial, conducted group interviews.

The interviews were conducted at the Physiotherapy in Women’s Health Research Unit, University of Alcalá, Madrid, Spain, and audio-recorded with participants’ permission.

### Data analysis

#### Quantitative statistical analysis

To evaluate the effects of the intervention on the continuous outcomes (VAS, SPADI, FACT-Bv4, RMS%) at assessment *t*, we used a separate multiple linear regression model adjusted by baseline (Eq. 1). This approach was chosen as it allows an efficient comparison of between-group differences at each follow-up time point while adjusting for baseline values, providing a straightforward interpretation of treatment effects.

1 $$(Y_{it}-Yi0)\:=\:\beta1tGi1\:+\:\beta2tGi2\:+\:\beta3tGi3\:+\:\beta4t\ast C(Yi0)\:+\:e_{it}$$

where *Y*_ij_ is the value of the outcome for individual “i” at assessment “t” (*t* = 0 at baseline, *t* = 1 after post-intervention, *t* = 2 at 3-month follow-up, and *t* = 3 at 6-month follow-up) and *C*(Yi0) is value of Yi0 centered on its mean. Gik is binary variable that takes value Gik = 1 when participant “i” belongs to group “k,” coding *k* = 1 for *Multimodal Physiotherapy*, *k* = 2 for *Scapular Exercises* only, and *k* = 3 for *Conventional Shoulder Exercises*. βkt is the mean change in *Y* in group “k” from assessment 0 to “t” in a “typical” individual with baseline measurement of *Y* equal to the mean (Yi0 = Mean(Y_i0_)). *β*_4t_ captures the regression to the mean, and eit is a random individual error with normal distribution eit~*N* (0, σe). We did linear hypothesis tests to compare changes between the groups ((β1t = β2t), (β1t = β3t), (β2t = β3t)).

In addition, between-group effect sizes were quantified using Cohen’s *d*, calculated as the difference between the expected mean differences divided by the standard deviation of the model errors.

To evaluate the effects of the intervention on the binary outcome of *myofascial pain syndrome*, we used a linear model for proportions depending on group and assessment and the interaction between the two (Eq. 2).

2 $${Y}_{it}=\sum_{t=0:3}{\beta}_{1t}{V}_{it}+ \sum_{k=1:3}{\beta}_{2k}{G}_{ik}+\sum_{k=1:3}{\beta}_{3tk}{V}_{it}{G}_{ik}+{e}_{it}$$

where *Y*_it_ is the outcome for individual “i” in assessment “t,” and Vit and Gik are binary variables that take values Gik = 1 when participant “i” belongs to group “k” and Vit = 1 in assessment “t.” Using appropriate combinations of the *β* coefficients, we can estimate the proportion of participants with the outcome in each group at each assessment. We found that this linear model would estimate correctly the observed proportions while logistic models often fail to converge due to the extreme distribution of the outcome in some assessments.

All statistical analyses were performed using R version 4.2.0 (R Foundation for Statistical Computing, Vienna, Austria).

#### Qualitative analysis

Four researchers manually transcribed the recorded interviews. Each transcript was assigned a code according to the intervention group, type of interview (individual or group), and participant order.

A triangulation process was carried out in which three researchers independently analyzed the transcripts. Through an iterative process, the researchers identified initial codes, discussed their meaning, and subsequently organized them into themes.

Coding and data management were conducted using ATLAS.ti version 6.1 software (Scientific Software Development GmbH, Berlin, Germany).

## Results

**Fig. 1:**
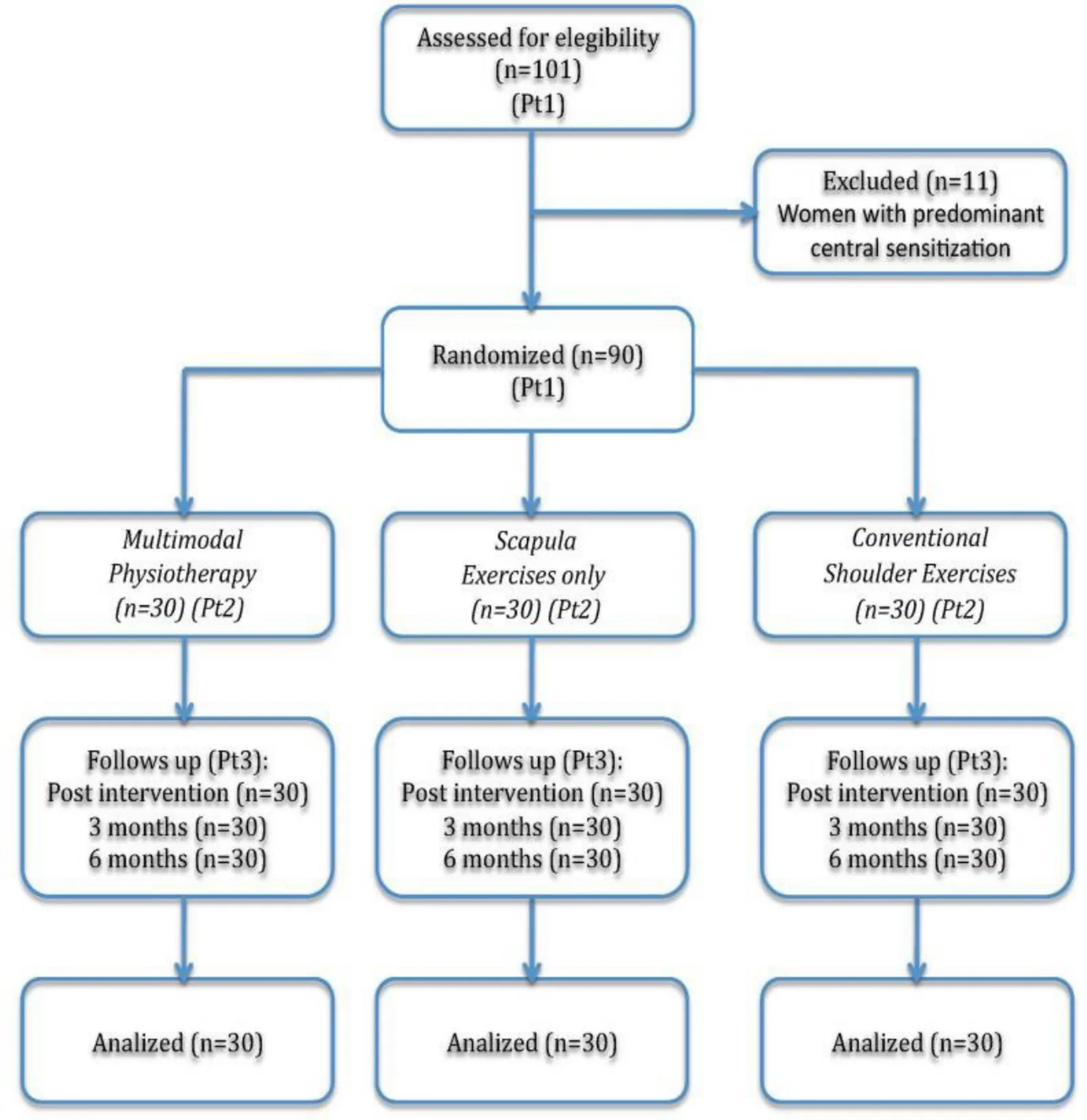
Flow diagram of participants through the study

A total of 90 participants were randomized (30 per group). All participants completed the intervention and follow-up assessments and were included in the analyses. Participant flow is presented in Fig. 1 in accordance with CONSORT guidelines. Clinical and demographic baseline characteristics are shown in Table 1. Adjusted within-group changes from baseline and between-group comparisons at each assessment point are presented in Tables 2 and 3. Effect sizes with *p*-values are reported for continuous outcomes (Table 2), and changes in proportion with *p*-values are reported for the binary outcome of MPS (Table 3).

**Table 1:** Participant baseline characteristics

Outcomes % or mean (SD) shown in the cells	MP (*n* = 30)	SE (*n* = 30)	CSE (*n* = 30)	Total sample (*n* = 90)
Age years	56.2 (9.7)	58.3 (7.1)	56.6 (8.9)	57 (8.6)
Body mass index (kg/m^2^)	24.7 (3.8)	25.7 (5.1)	23.1 (5.5)	24.5 (4.9)
Similar dominance and treatment side:	50%	40%	47%	46%
Surgical procedure:				
Quadrantectomy	80%	83%	93%	86%
Lumpectomy	20%	17%	7%	14%
Postoperative therapy:				
Chemotherapy	97%	100%	97%	98%
Radiotherapy	100%	100%	100%	100%
Hormonal therapy	73.0%	77%	73%	74%
Serome	0	3%	0	1%
History of axillary web syndrome	90%	87%	87%	88%
Lymphocele	13%	3%	3%	7%
Scapular dyskinesia:				
Pattern 1	13%	13%	13%	13%
Pattern 2	20%	17%	17%	18%
Pattern 3	10%	10%	20%	13%
Pattern 5	57%	60%	50%	56%
Abnormal scapular static position:				
Internal rotation	97%	90%	87%	91%
Anterior tilt	87%	93%	97%	92%
Downward rotation	47%	50%	53%	50%
Abduction	23%	17%	27%	22%
Characteristics of pain:				
VAS	7.5 (1.5)	7.6 (2.9)	7.1 (1.3)	7.4 (1.3)
Pain duration (months)	9.07 (1.74)	9.30 (1.93)	8.97 (1.83)	9.11 (1.82)
Predominant neuropathic pain	83%	76%	93%	84%
Mix pain mechanisms	100%	100%	100%	100%
Positive ULNT1	100%	100%	100%	100%
Other outcomes:				
SPADI	73.8 (3.1)	73.0 (1.7)	73.2 (2.7)	73.3 (2.9)
FACT-Bv4	74.1 (5.0)	75.1 (5.4)	74.1 (5.3)	74.4 (5.2)
RMS% UT	54.2 (10.3)	55.0 (10.9)	60.2 (10.9)	56.5 (10.9)
RMS% LT	10.9 (4.8)	11.6 (4.8)	10.9 (4.6)	11.2 (4.7)
RMS% MD	80.6 (9.3)	78.6 (9.9)	80.4 (14.2)	79.9 (11.3)
RMS% I	6.7 (3.1)	7.4 (2.5)	6.4 (2.3)	6.9 (2.7)
RMS% SA	5.2 (2.5)	5.9 (2.3)	5.9 (2.3)	5.7 (2.4)
MPS	100%	100%	100%	100%

Summary statistics are mean (standard deviation) or percentages

*MP* multimodal physiotherapy, *SE *scapular exercises, *CSE* conventional shoulder exercises, *ULNT1* Upper Limb Neural Tension Test (positive); scapular dyskinesis test: pattern 1 (anterior tilt), pattern 2 (winging), pattern 3 (dysrhythmia), pattern 4 (normal scapulohumeral rhythm), pattern 5 (mix pattern), *VAS* Visual Analog Scale, *SPADI* total score of Shoulder Pain and Disability Index Questionnaire, *FACT-Bv4* total score of Functional Assessment of Cancer Therapy-Breast Questionnaire (version 4), *RMS% UT* root mean square % of upper trapezius muscle, *RMS% LT* root mean square % of lower trapezius muscle, *RMS % I* root mean square % of infraspinatus muscle, *RMS% MD* root mean square % of middle deltoid muscle, *RMS% SA* root mean square % of serratus anterior muscle, *MPS* myofascial pain syndrome

**Table 2:** Adjusted within-group changes from baseline and between-group effect sizes for continuous outcomes across assessments

var	A	Intra-group changes from baseline assessment: adjusted mean difference (95%CI)			Between-groups comparison at each assessment: effect size (*p*-values)		
		MP*	SE*	CSE*	MP–SE **	MP–CSE**	SE–CSE**
VAS (cm)	A1	−5.6 (−5.9 to −5.3)	−3.9 (−4.2 to −3.6)	−0.6 (−0.9 to −0.3)	−2.12 (*p* < 0.001)	−6.27 (*p* < 0.001)	−4.15 (*p* < 0.001)
VAS (cm)	A2	−5.9 (−6.2 to −5.6)	−4.5 (−4.8 to −4.2)	−0.4 (−0.7 to −0.0)	−1.65 (*p* < 0.001)	−6.50 (*p* < 0.001)	−4.85 (*p* < 0.001)
VAS (cm)	A3	−6.3 (−6.7 to −5.9)	−5.7 (−6.0 to −5.3)	−0.3 (−0.7 to 0.0)	−0.63 (*p* < 0.045)	−5.71 (*p* < 0.001)	−5.08 (*p* < 0.001)
SPADI	A1	−44.6 (−45.7 to −43.4)	−22.7 (−23.8 to −21.5)	−8.4 (−9.5 to −7.2)	−6.93 (*p* < 0.001)	−11.46 (*p* < 0.001)	−4.53 (*p* < 0.001)
SPADI	A2	−58.5 (−59.9 to −57.1)	−47.1 (−48.5 to −45.7)	−15.7 (−17.1 to −14.4)	−3.00 (*p* < 0.001)	−11.25 (*p* < 0.001)	−8.25 (*p* < 0.001)
SPADI	A3	−60.9 (−62.3 to −59.5)	−53.5 (−54.9 to −52.1)	−18.7 (−20.1 to −17.4)	−1.95 (*p* < 0.001)	−11.15 (*p* < 0.001)	−9.20 (*p* < 0.001)
FACTBv4	A1	11.7 (10.6 to 12.8)	6.4 (5.3 to 7.5)	1.8 (0.6 to 2.9)	1.75 (*p* < 0.001)	3.27 (*p* < 0.001)	1.52 (*p* < 0.001)
FACTBv4	A2	19.8 (18.3 to 21.3)	15.0 (13.5 to 16.5)	6.0 (4.5 to 7.5)	1.15 (*p* < 0.001)	3.32 (*p* < 0.001)	2.16 (*p* < 0.001)
FACTBv4	A3	22.5 (20.8 to 24.2)	17.0 (15.3 to 18.7)	8.9 (7.2 to 10.6)	1.18 (*p* < 0.001)	2.92 (*p* < 0.001)	1.75 (*p* < 0.001)
RMS%UT	A1	−12.9 (−14.1 to −11.6)	−6.1 (−7.3 to −4.9)	−2.5 (−3.8 to −1.3)	−1.99 (*p *< 0.001)	−3.05 (*p* < 0.001)	−1.05 (*p* < 0.001)
RMS%UT	A2	−20.1 (−21.6 to −18.6)	−11.3 (−12.8 to −9.8)	−4.0 (−5.5 to −2.5)	−2.15 (*p* < 0.001)	−3.93 (*p* < 0.001)	−1.78 (*p* < 0.001)
RMS%UT	A3	−25.5 (−27.1 to −23.9)	−15.2 (−16.8 to −13.6)	−5.6 (−7.3 to −4.0)	−2.35 (*p* < 0.001)	−4.55 (*p* < 0.001)	−2.20 (*p* < 0.001)
RMS% LT	A1	5.7 (5.1 to 6.3)	4.1 (3.5 to 4.7)	1.3 (0.7 to 1.9)	0.97 (*p* < 0.001)	2.63 (*p* < 0.001)	1.67 (*p* < 0.001)
RMS%LT	A2	10.7 (9.7 to 11.7)	7.7 (6.7 to 8.7)	2.0 (1.0 to 3.0)	1.07 (*p* < 0.001)	3.13 (*p* < 0.001)	2.06 (*p* < 0.001)
RMS%LT	A3	17.0 (15.7 to 18.4)	11.2 (9.9 to 12.5)	2.3 (1.0 to 3.6)	1.62 (*p* < 0.001)	4.08 (*p* < 0.001)	2.46 (*p* < 0.001)
RMS%MD	A1	−7.8 (−9.5 to −6.2)	−5.8 (−7.5 to −4.2)	−4.2 (−5.9 to −2.6)	−0.43 (*p* < 0.224)	−0.79 (*p* < 0.008)	−0.36 (*p* < 0.348)
RMS%MD	A2	−14.8 (−16.4 to −13.1)	−9.9 (−11.6 to −8.2)	−5.8 (−7.4 to −4.1)	−1.06 (*p* < 0.001)	−1.96 (*p* < 0.001)	−0.90 (*p* < 0.002)
RMS%MD	A3	−21.5 (−23.3 to −19.6)	−13.7 (−15.6 to −11.9)	−7.0 (−8.9 to −5.1)	−1.48 (*p* < 0.001)	−2.78 (*p* < 0.001)	−1.30 (*p* < 0.001)
RMS%I	A1	3.0 (2.0 to 4.0)	1.9 (0.9 to 2.8)	3.4 (2.4 to 4.4)	0.42 (*p* < 0.243)	−0.15 (*p* < 0.821)	−0.58 (*p* < 0.077)
RMS%I	A2	7.3 (6.7 to 7.8)	5.0 (4.4 to 5.6)	1.9 (1.3 to 2.4)	1.41 (*p* < 0.001)	3.37 (*p* < 0.001)	1.96 (*p* < 0.001)
RMS%I	A3	9.8 (9.1 to 10.5)	7.0 (6.3 to 7.7)	2.4 (1.7 to 3.1)	1.41 (*p* < 0.001)	3.78 (*p* < 0.001)	2.37 (*p* < 0.001)
RMS%SA	A1	3.5 (3.1 to 3.9)	2.3 (2.0 to 2.7)	1.4 (1.1 to 1.8)	1.15 (*p* < 0.001)	2.04 (*p* < 0.001)	0.89 (*p* < 0.003)
RMS%SA	A2	6.5 (6.0 to 7.0)	4.5 (4.0 to 5.0)	2.1 (1.6 to 2.6)	1.41 (*p* < 0.001)	3.13 (*p* < 0.001)	1.72 (*p* < 0.001)
RMS%SA	A3	7.9 (7.4 to 8.5)	6.1 (5.5 to 6.7)	2.3 (1.7 to 2.9)	1.17 (*p *< 0.001)	3.60 (*p* < 0.001)	2.43 (*p* < 0.001)

*A* assessment, *A1* post-intervention assessment, *A2* 3-month follow-up post-intervention, *A3* 6-month follow-up post-intervention, *VAS* Visual Analog Scale, *SPADI* total score of Shoulder Pain and Disability Index Questionnaire, *FACT-Bv4* total score of Functional Assessment of Cancer Therapy-Breast Questionnaire (version 4), *RMS% UT* root mean square % of upper trapezius muscle, *RMS% LT* root mean square % of lower trapezius muscle, *RMS % I* root mean square % of infraspinatus muscle, *RMS% MD* root mean square % of middle deltoid muscle, *RMS% SA* root mean square % of serratus anterior muscle, *MP* multimodal physiotherapy, *SE* scapular exercises, *CSE* conventional shoulder exercises

*In these columns, we show the intra-group expected mean difference (with its confidence interval) from baseline visit 0, in a regression model adjusted by baseline value of the variable

**In these columns, we show the Cohen’s *d* effect size between groups as differences between the expected mean differences divided by the standard deviation of the errors in the model (and the *p*-value of that comparison)

**Table 3:** Adjusted within-group changes from baseline and between-group comparisons for the binary outcome across assessments

var	A	Intra-group changes from baseline assessment: adjusted difference in proportion (95% CI)			Between-group comparisons at each assessment (*p*-values)		
		MP*	SE*	CSE*	MP–SE**	MP–CSE**	SE–CSE**
MPS	A1	−80% (−100 to −60)	−20% (−40 to 0)	0% (−20 to 20)	< 0.001	< 0.001	0.294
MPS	A2	−90% (−100 to −70)	−70% (−90 to −50)	−10% (−30 to 10)	0.034	< 0.001	< 0.001
MPS	A3	−90% (−100 to −70)	−80% (−100 to −60)	−20% (−40 to 0)	0.361	< 0.001	< 0.001

*A* assessment, *A1* post-intervention assessment, *A2* 3-month follow-up post-intervention, *A3* 6-month follow-up post-intervention, *MPS* myofascial pain syndrome, *MP* multimodal physiotherapy, *SE* scapular exercises, *CSE* conventional shoulder exercises

*These columns show the within-group adjusted changes from baseline in the estimated proportion of participants with MPS, with 95% confidence intervals, based on a regression model

**These columns show the between-group comparisons at each assessment (*p*-values)

### Pain intensity (VAS)

**Fig. 2:**
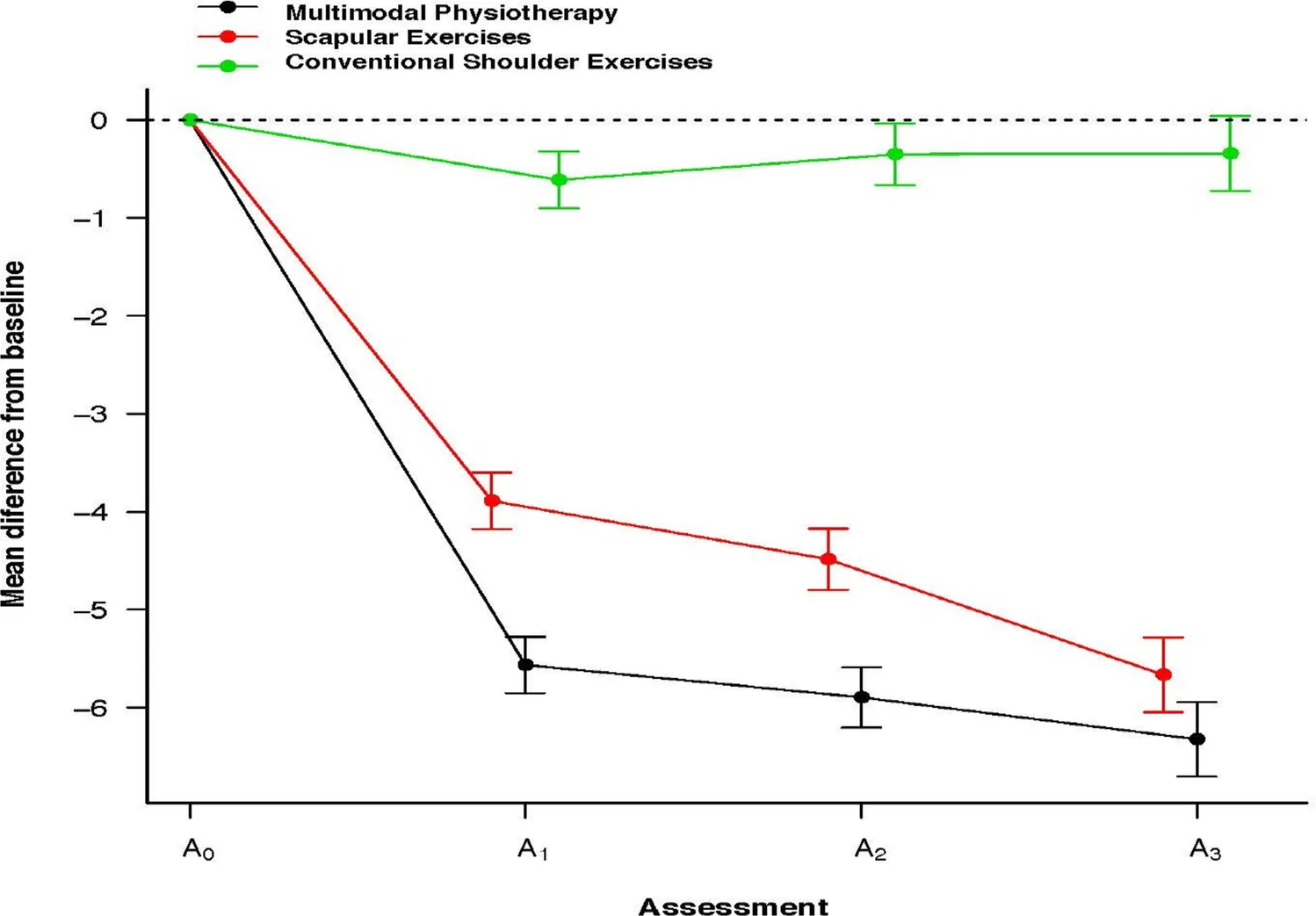
Evolution of subjective pain intensity (VAS pain) throughout this study in the three groups showing the effects in each group at post-intervention (A_1_), 3-month (A_2_), and 6-month (A_3_) follow-up

Pain intensity (primary outcome) decreased over time in all groups, with greater and more consistent reductions observed in the MP and SE groups compared with the CSE group (Fig. 2; Table 2).

Between-group comparisons showed that MP resulted in significantly greater pain reduction than SE at all assessment points, with large effects at post-intervention and 3-month follow-up, and a moderate effect at 6 months (Table 2). Both MP and SE were superior to CSE across all time points, consistently showing large between-group effects.

Clinically meaningful improvements (exceeding the MCID) were observed in both MP and SE groups at all assessment points, whereas the CSE group did not reach the MCID at any time point. Overall, MP demonstrated the greatest reduction in pain, followed by SE, while CSE showed limited clinical impact.

### Perceived shoulder pain and disability (SPADI)

SPADI scores decreased progressively over time in all groups (Table 2), with the greatest reductions observed in the MP group, followed by SE and then CSE.

Between-group differences were statistically significant at all assessment points, with large effect sizes favoring MP over both SE and CSE and SE over CSE.

Clinically meaningful improvements (exceeding the MCID) were observed in the MP and SE groups at all assessment points. In contrast, the CSE group reached the MCID only at the 6-month follow-up.

### Health-related quality of life (FACT-Bv4)

FACT-Bv4 scores increased progressively over time in all groups (Table 2), with greater improvements observed in the MP group, followed by SE and then CSE.

Between-group differences were statistically significant at all assessment points, with large effect sizes favoring MP over both SE and CSE and SE over CSE.

Clinically meaningful improvements were observed in the MP group at all assessment points. In the SE group, clinically meaningful improvements were observed at all time points except immediately post-intervention, whereas in the CSE group, the MCID was reached only at the 6-month follow-up.

### Muscle activation (RMS%)

Overall, muscle activation patterns improved over time in all groups, with greater and more consistent changes observed in the MP group, followed by SE and then CSE (Table 2).

For the upper trapezius and middle deltoid, RMS% decreased across all groups, with significantly greater reductions in the MP group at all assessment points. Between-group differences were statistically significant across follow-up assessments, with large effects favoring MP over both SE and CSE and SE over CSE.

For the lower trapezius and serratus anterior, RMS% increased progressively over time in all groups, with significantly greater increases in the MP group at all assessment points. SE also demonstrated greater improvements than CSE across all time points.

For the infraspinatus, increases in RMS% were observed in the MP and SE groups, whereas the CSE group showed smaller changes that did not progress over time. Between-group differences were not statistically significant immediately post-intervention but became significant at the 3- and 6-month follow-ups, with large effects favoring MP and SE over CSE.

Overall, MP produced the most pronounced improvements in muscle activation, followed by SE, while CSE showed more limited changes.

### Myofascial pain syndrome

At baseline, all participants presented with MPS (100%). A significant reduction in the proportion of participants with MPS was observed in the MP group from post-intervention onwards (Table 3).

In the SE group, significant reductions were observed from the 3-month follow-up, whereas no statistically significant reduction was observed in the CSE group at any assessment point.

Between-group comparisons showed that MP was superior to SE at post-intervention and 3 months, with no significant difference at 6 months. MP was superior to CSE at all assessment points, while SE did not differ from CSE immediately post-intervention but was superior at 3 and 6 months.

### Exercise adherence

Exercise adherence following the supervised intervention is presented in Table 4. Participants showed moderate adherence to the prescribed home exercise program, with adherence rates of 68% at 3 months and 55% at 6 months.

**Table 4:** Adherence to the home exercises

		MP	SE	CSE	Total sample
Adherence %	A2	83%	73%	47%	68%
	A3	77%	67%	40%	55%

*A* assessment, *A1* post-intervention assessment, *A2* 3-month follow-up post-intervention, *A3* 6-month follow-up post-intervention, *MP* multimodal physiotherapy, *SE* scapular exercises, *CSE* conventional shoulder exercises

Descriptively, adherence was higher in the MP and SE groups than in the CSE group.

### Interviews

Four participants from each intervention group participated in the qualitative component of the study. In total, two focus group interviews and three individual interviews were conducted, with an average duration of 44.6 min.

Five main thematic categories were identified through thematic analysis: (1) personal experience of treatment-related side effects, (2) professional management of treatment-related side effects , (3) perceived benefits of the physiotherapy program, (4) integration of exercises into daily life, and (5) return to normal life (Supplementary data C).

Participants identified pain reduction and perceived effectiveness as key facilitators of adherence:

"Doing them or not doing them makes a huge difference for me".

The acquisition of knowledge and the reconceptualization of pain were also highlighted as important factors supporting self-management:

"I learned to identify when and why the pain appeared, and then I applied the exercises".

Barriers to adherence included competing daily demands, difficulty integrating exercises into daily routines, and the desire to move away from the disease experience. Additional thematic descriptions and representative quotations are provided in Supplementary data C.

## Discussion

The increased success of breast cancer treatment has led to an increase in the overall survival rate in women to approximately 78–88% at 5 years [45]. Nevertheless, many patients continue to experience secondary effects derived from treatment including pain and disability [45]. Pain is one of the most significant complications contributing to reduced satisfaction and quality of life [45]. PPBCT represents a major survivorship issue affecting long-term function and quality of life.

Pain intensity was the primary outcome of this study, and the pattern of within-group changes alongside between-group effect sizes offers important insights into the differential mechanisms driving recovery across interventions. Both the MP and SE groups achieved progressive and clinically meaningful reductions in pain across all assessment points, consistently exceeding the MCID. The large between-group effect size observed immediately after the intervention (d = −2.12; *p* < 0.001), which remained large at 3 months and decreased to a small-to-moderate effect at 6 months (*d* = −0.63, *p* = 0.045), suggests that scapular exercise-based rehabilitation may represent a key component for long-term recovery, while the addition of other techniques may primarily contribute to earlier symptom reduction. These findings highlight the temporal differences in treatment effects across interventions, with MP providing earlier benefits and exercise-based approaches contributing to sustained improvements. The early reduction of pain in response to the MP intervention may be attributed to the inclusion of different techniques designed to address dysfunction in muscle, neural, and myofascial tissues. Furthermore, it is important to emphasize that this faster pain reduction may be beneficial, as it may contribute to decreasing peripheral sensitization, which could potentially reduce the risk of developing nociplastic pain [3].

The pain reduction observed in the CSE group may be insufficient to achieve clinically meaningful improvement, as it did not reach the MCID at any assessment point. The consistently large between-group effect sizes relative to both active interventions reinforce this interpretation.

Regarding the pain characterization of the women included in this study, all presented mixed pain mechanisms, with 84% of the participants showing predominantly neuropathic pain, in agreement with previous findings [3, 4]. A recent study conducted in 86 breast cancer survivors, based on the CANPPHE Network multidisciplinary international guidelines, showed that the most frequent phenotype was mixed pain (40.70%), whereas predominantly nociplastic pain accounted for 16.28% of the cases [4]. In line with these findings, our study explicitly excluded cases with a predominantly nociplastic pain phenotype, as this type of pain requires a more complex and specialized therapeutic approach [3, 4, 15]. By excluding this subgroup, we aimed to avoid a sample dominated by this specific clinical profile, which is less represented within the spectrum of PPBCT, allowing us to focus on a more homogeneous group of women.

Treatment of breast cancer can lead to alterations in neural tissue resulting in increased mechanosensitivity of peripheral nerves [13, 14, 35]. Accordingly, in this study, all participants presented a positive ULNT1, and those within the multimodal physiotherapy group received neurodynamic mobilization. The methodological design of this study does not allow for the determination of the specific contribution of neurodynamic mobilization or deep dry needling. Therefore, the observed improvements should be interpreted as the result of the combined intervention. However, previous studies support the efficacy of neurodynamic mobilization in relation to disability and pain [18, 39, 46], even up to 1 year after surgery [46].

All women presented active MTrPs, which may be a further consequence of peripheral sensitization [1, 12]. Although the percentage of participants presenting with active MTrPs gradually decreased in all three groups, the reduction was significantly greater following multimodal physiotherapy starting at A1. This supports the addition of deep dry needling to therapeutic exercise for the treatment of active MTrPs. Nevertheless, no significant differences were observed at A3 between the MP group and the SE group, which could be related to the role of scapular positioning and movement coordination in shoulder mechanics, as well as the improvement of movement patterns [9–11]. In addition to these findings, no adverse events, including lymphedema, were reported during the study. While this supports the clinical feasibility of including dry needling within an MP program in this population, these results should be interpreted with caution, as the study was not specifically designed to evaluate safety outcomes. The application of dry needling followed strict screening criteria, aseptic procedures, and was delivered by an experienced clinician, which is consistent with current recommendations for invasive physiotherapy techniques [16]. Furthermore, recent evidence suggests that dry needling is among the non-pharmacological interventions showing potential benefit in the management of postmastectomy pain syndrome, although the available evidence remains limited and heterogeneous [47]. Previous studies in patients with post-mastectomy pain have also reported its use under controlled conditions without serious adverse events [48]. It is also important to consider that needling techniques in the thoracic region may involve potential risks, such as pneumothorax, although these are not specific to oncological populations, reinforcing the need for appropriate clinical reasoning and precautionary measures [49]. These findings should be interpreted within the context of a controlled clinical environment and may not be generalizable to all clinical settings.

The participants displayed alterations in the static position of their scapula, as well as poor control of their scapula muscles when specifically tested. Increased scapular anterior tilt and internal rotation were commonly observed. These findings are consistent with another descriptive study conducted in women with the same clinical profile [11]. Regarding muscle activation (RMS%), a progressive improvement was observed in the activation of the lower trapezius, serratus anterior, and infraspinatus across all groups, with the greatest improvements seen in the MP group, followed by the SE group. Although at the first assessment point (A1) between-group differences were not always significant for some muscles, significant differences were already observed in others, particularly in the lower trapezius and serratus anterior, favoring the MP group. As the follow-up progressed (A2 and A3), MP demonstrated clearer superiority, with statistically significant differences and large effect sizes, especially for the lower trapezius and serratus anterior compared to the SE and CSE groups. Scapular exercises also showed improvements, but of lesser magnitude compared to MP. However, SE were more effective than CSE, showing consistent improvements across most assessment points.

In terms of the middle deltoid and upper trapezius activation, both showed progressive improvement in all groups, with the greatest reduction in activation observed in the MP group. Although differences in middle deltoid activation were smaller at earlier assessment points (A1), they became significantly more pronounced from 3 months (A2) up to 6 months (A3), with large effect sizes favoring MP over scapular exercises and CSE.

These patterns of reduced activation of superficial muscles and increased activation of deeper stabilizers, such as the serratus anterior and lower trapezius, may reflect improved scapular muscle coordination and more efficient motor patterns, rather than indicating a direct normalization of biomechanics [9–11]. These trends suggest that, although SE are effective, MP appears to offer a greater advantage, particularly in the medium and long term, in improving muscle activation and scapular function [9–11].

The results show that MP had a significantly greater impact on pain and disability improvement (SPADI), with consistently large effect sizes across all assessment points, surpassing the SE group at all assessment points. These improvements were clinically significant, exceeding the MCID at all points. While SE also showed improvements above the MCID, these were smaller than those observed with MP. On the other hand, SE were more effective than CSE, with clinically meaningful improvements at all assessment points. These findings indicate that, although scapular exercises are relevant, they appear to be less effective than multimodal physiotherapy when used as a standalone intervention for women with PPBCT, highlighting the potential added value of a multimodal approach.

The superiority of SEs over CSE, and their added value within the MP group in improving pain and function (measured with VAS, SPADI, and RMS%), can be explained not only by the crucial role of the scapula in shoulder dynamics [9–11], but also by the inclusion of scapular motor control exercises. The goal was not only to improve muscle activation but also to modify pain-related memories and maladaptive motor responses [11, 20, 50, 51]. Additionally, the aim was to enhance movement quality and reduce pain-related avoidance behaviors through cognition-oriented training and gradual exposure to movement [11, 20, 50, 51]. Therefore, the scapular motor control exercises, as implemented in our program, are not merely low-load alternatives to strength and endurance training, but rather structured exposures to controlled movement, with particular attention to movement quality, sequencing, and progressive load tolerance, in line with current evidence and clinical recommendations supporting multimodal and exercise-based interventions for the management of persistent musculoskeletal pain [11, 20, 50, 51].

The FACT-Bv4 scale was used to evaluate health-related quality of life and at baseline all participants presented with relatively low scores, consistent with previous reports evaluating health-related quality of life in women treated for breast cancer, especially after surgery [45]. Participants receiving MP showed significant and clinically meaningful improvements in health-related quality of life, which were consistently greater than those observed in the SE and CSE groups across all post-intervention assessments. These between-group differences were associated with large effect sizes.

Furthermore, SE demonstrated greater effectiveness than CSE at all assessment points, with clinically meaningful improvements in the medium and long term. These findings are consistent with the improvements observed in pain and physical functioning, suggesting that MP and to a lesser extent SE may positively influence both emotional and physical well-being.

Exercise adherence is essential, especially to maintain health-related quality of life, and participants in this study showed moderate adherence (A2: 68%; A3: 55%) to their home exercise program. Differences in adherence between groups were observed, with lower adherence in the CSE group. These findings should be considered when interpreting the overall pattern of results, although no causal relationship can be established.

As reflected in the qualitative findings, perceived effectiveness and pain reduction were identified as key facilitators of adherence. The integration of exercises into daily routines and the reconceptualization of pain also played an important role, whereas competing life demands and the desire to move away from the disease experience were identified as barriers.

These findings help to contextualize the observed adherence patterns, particularly the lower adherence in the conventional shoulder exercise group, which experienced less symptom improvement. Together, they highlight the importance of selecting effective and meaningful exercises, as well as implementing strategies that facilitate their integration into daily life.

In addition, the role of pain reconceptualization and active patient involvement identified in this study is consistent with previous literature on chronic pain management, which emphasizes the importance of addressing maladaptive beliefs and promoting self-management strategies [52, 53].

### Methodological considerations

A possible limitation of this study was the difficulty in controlling external interventions, such as the use of anti-inflammatory or analgesic drugs. At each assessment point, participants were instructed about this, and we checked whether any medication use or additional physiotherapy treatment had occurred. No additional external interventions were reported.

Furthermore, EMG normalization based on maximal or submaximal contractions [30] may be challenging in individuals with pain. For this reason, EMG amplitude was normalized to baseline activity [11]. Additionally, the exclusion of women unable to achieve 90° of shoulder elevation, a criterion necessary to ensure the feasibility of the standardized sEMG assessments, as well as those with active lymphedema requiring treatment, should be considered when interpreting the results, particularly with regard to the external validity of the findings in relation to the broader PPBCT population.

Despite these limitations, this study also has several methodological strengths. The trial combined patient-reported outcomes with objective neuromuscular measures (sEMG) and a qualitative exploration of exercise adherence. This multidimensional approach provides a more comprehensive evaluation of physiotherapy interventions in women with PPBCT, capturing not only symptom changes but also neuromuscular adaptations and patient experiences related to long-term exercise adherence.

## Conclusions

Multimodal physiotherapy combining scapular exercises, deep dry needling, and neurodynamic techniques was more effective than exercise programs alone in improving clinical outcomes in women with PPBCT. Scapular exercise-based physiotherapy appears to play a key role in long-term recovery, while the addition of other physiotherapy techniques may primarily contribute to earlier symptom reduction, without adverse events such as lymphedema. 

These findings support the use of a multimodal approach to address the complex mechanisms underlying persistent pain following breast cancer treatment. In addition, qualitative findings suggest that long-term adherence may be enhanced by exercise programs that include effective strategies easily integrated into daily life.

## Supplementary Information

Below is the link to the electronic supplementary material.

ESM 1 (DOCX 3.57 MB)

## Data Availability

The datasets generated and analysed during the current study are not publicly available due to privacy and ethical restrictions but are available from the corresponding author on reasonable request.
