# Supplementary material for: Multimodal physiotherapy versus scapular and conventional exercise for persistent pain following breast cancer treatment: a randomized controlled trial
Source: J Cancer Surviv. 2026 May 23;20(Suppl 2):2096–111. doi: 10.1007/s11764-026-02035-z (PMC13744458; doi:10.1007/s11764-026-02035-z)
Supplement: Supplementary file 1 [file 11764_2026_2035_MOESM1_ESM.docx]

**Supplementary data A**

## SCAPULAR EXERCISES

A. **Scapular motor training with emphasis on a scapular orientation exercise (SOE)**

**Step 1**. Conscious correction of scapular orientation was taught using visual, auditory, and kinesthetic cues (figure Ia).

**Step 4.** Functional upper limb exercises with progressive overload were included.

**Step 2.** Women carried out these exercises by themselves. Exercise holding time and number of repetitions were increased progressively

**Step 3**. Women practiced this exercise until satisfactory correction (it had to be held for at least 10 seconds and 10 repetitions without help).

## B. Training of scapular stabilizing muscles

Ten repetitions of each exercise were performed with an emphasis on obtaining a neutral scapular orientation. Furthermore, these exercises were carried out with progressive load, using dumbbells and elastic bands. The exercises were:

1. **Wall slide.** Stand facing wall in staggered stance with dominant foot at the base of the wall. Ulnar portion of forearms in contact with wall with shoulder and elbow flexed to 90º; the participant was instructed to slide the forearms up, and down the wall.

2. **Side lying forward flexion.** Starting with the arm positioned along the body, the patient performed 90° of forward flexion in the sagittal plane.

3. **Side lying external rotation.** Starting with the shoulder in a neutral position and the elbow flexed to 90°, the patient performed external rotation of the shoulder with a pillow between the elbow and trunk to avoid compensatory movements.

**Supplementary data B**

## CONVENTIONAL SHOULDER EXERCISES

**A. Shoulder mobilization exercises (ten repetitions)**

1). Forward flexion starts with the shoulders alongside body in a supine position.

2). Bilateral shoulder circles in sitting position.

## B) Stretching exercises (ten repetitions/ holding for fifteen seconds)

1) The patient positioned their affected shoulder at 90-degree forward flexion and placed their other hand on the outside of the elbow of the affected side to perform horizontal shoulder adduction.
2) They positioned their affected shoulder at180-degree forward flexion and placed their other hand on the outside of the elbow of the affected side to perform a contralateral trunk side bend.
3) Standing with the side of the body of the affected shoulder towards the wall and the palm of the hand placed on the wall with the 90-degree shoulder abduction. They then rotated their feet and trunk 45 degrees away from the wall to create a stretch across the upper chest.

# 1 2 3

## C) Isometric shoulder exercises (ten repetitions)

1) Performed in standing facing a wall, isometric shoulder flexion was performed with the elbow flexed to 90 degrees, pressing for five seconds.
2) Carried out in standing with their back facing a wall, isometric shoulder extension was performed with the elbow flexed	to	90 degrees, pressing the wall for five seconds.
3) Performed in standing, with the body facing the outside corner of a wall, isometric shoulder internal rotation was performed with the elbow flexed to 90 degrees, pressing the wall for five seconds.
4) Carried out in standing, with the body perpendicular to a wall, isometric shoulder external rotation was performed with the elbow flexed to 90 degrees, pressing the wall for five seconds.

# 1 2 3 4

## D) Resistance bands shoulder exercises (ten repetitions)

1) Performed in standing, with their ipsilateral foot over a resistance band, they flexed her shoulder until 90º.

2) Performed	in standing, with	their contralateral	foot	over	the	resistance band, they performed shoulder	abduction	until	reaching	90 º.

3) Carried	out	in standing,	with	their ipsilateral	foot	over	the	resistance	band, they performed	shoulder	adduction	until	reaching	90º.

4) Performed in standing, with the unaffected shoulder perpendicular to a wall and the elbow flexed to 90º, they performed shoulder external rotation until reaching 30-60º using an elastic band tied to the doorknob.

5) Carried out in standing, with the affected shoulder perpendicular to a wall and the elbow flexed to 90 degrees, they performed shoulder internal rotation until reaching 30-60 degrees using an elastic band tied to the doorknob.

In addition, all participants were instructed to perform their respective exercises three days per week throughout the follow-up period.

**Supplementary Data C. Qualitative findings on adherence to home-based exercises after physiotherapy intervention**

**Qualitative overview**

Semi-structured individual interviews and focus groups explored participants’ experiences regarding adherence to home-based therapeutic exercises after completion of the intervention. Five main thematic categories were identified through thematic analysis: (1) personal experience of treatment-related side effects, (2) professional management of treatment-related side effects, (3) perceived benefits of the physiotherapy program, (4) integration of exercises into daily life, and (5) return to normal life. Representative quotations are presented below together with the thematic synthesis shown in Supplementary Table C1.

**Theme 1. Personal experience of treatment-related side effects**

Participants described persistent pain, discomfort, fear of aggravating symptoms, and concern about developing long-term vascular or musculoskeletal sequelae after breast cancer treatment. These experiences frequently influenced daily activities and motivated behavioural adaptations intended to avoid symptom exacerbation.

DG2P1: “I try not to carry too much weight; I shift the load to the other side”.

Some women explained that symptom persistence reinforced adherence to the exercises, whereas others described avoidance behaviours linked to emotional distress and the desire to distance themselves from the cancer experience and its treatment.

DI1: “You have an illness, and you want to forget everything related to it. And that includes having to do things that remind you every day that you have had that illness”.

**Theme 2. Professional management of treatment-related side effects**

Participants highlighted inconsistencies in the information and recommendations received from healthcare professionals. The lack of consensus regarding symptom management sometimes generated uncertainty and reduced confidence in therapeutic strategies.

DG2P2: “All I want is to have the reconstruction done, to see if this discomfort in my rib goes away”.

Several participants perceived surgery or reconstruction as the definitive solution for symptoms, while others expressed difficulties identifying which recommendations were most appropriate.

**Theme 3. Perceived benefits of the physiotherapy program**

Participants described pain reduction and perceived effectiveness as major facilitators for maintaining exercise adherence. Improvement in symptoms reinforced motivation and encouraged continuation of the prescribed exercises.

DG1P1: “That is why I always do them — doing them or not doing them makes a huge difference for me”.

The acquisition of knowledge and reconceptualization of pain were also identified as important therapeutic benefits. Participants reported that pain education facilitated symptom interpretation, reduced anxiety, and improved self-management.

DG1P3: “I learned very well to identify when and what the reason for the pain was. And then, when I identify the pain, that is when I apply the exercises”.

Participants additionally reported that written materials and video recordings facilitated recall of exercises and recommendations, especially when some exercises were perceived as difficult or easy to forget.

**Theme 4. Integration of exercises into daily life**

The incorporation of exercises into daily routines emerged as an important facilitator of long-term adherence. Participants described developing personal strategies to integrate therapeutic exercises into everyday activities.

DG1P1: “I have got into the habit of doing them in the morning and at night”.

A sense of personal responsibility and active involvement in symptom management were also perceived as important factors supporting adherence. Some women explained that the intervention helped them identify safe movement strategies that allowed them to continue daily activities with less fear of triggering pain.

**Theme 5. Return to normal life**

Returning to work, resuming family responsibilities, and re-engaging in social and physical activities were frequently identified as barriers to maintaining the exercise program.

DI3: “At the gym, I don't do the resistance band exercise, for example, but there are some arm exercises done in a circuit that are very similar to the band one”.

Some women reported replacing the prescribed exercises with general physical activity, assuming both approaches were equivalent. Time constraints related to work and caregiving responsibilities also limited continuity of the exercises.

Family support emerged as a relevant facilitator, particularly when household responsibilities were redistributed.

DI1: “So, at home, I have redistributed the chores. The heavy tasks — well, my husband can do those.”

| **Supplementary Table C1.** Thematic categories, codes, and descriptions. | | |
| --- | --- | --- |
| **Family** | **Code** | **Description** |
| **Personal experience of the side effects of breast cancer treatment** | **Experiences of pain or unpleasant symptoms** | Identification of unpleasant symptoms experienced in their upper limb and affected side of the thorax after surgical treatment.  This includes the perception of pain, tingling, burning, numbness, punctures and  tightness. |
|  | **Other consequences** | Appearance or risk of suffering consequences other than pain in the upper limb ipsilateral to the affected side, including vascular issues (lymphedema, superficial lymphatic thrombosis) or musculoskeletal problems that produce limitations to movement. |
|  | **Psychological factors** | Referred to the desire to normalize their life. |
|  | **Wellness** | Perception of good general health status. |
| **Professional management of treatment-related side effects** | **Prevention** | Therapeutic approach that anticipates the appearance of symptoms and recurrence. |
|  | **Variety of exercises** | Indication of different therapeutic exercises. |
|  | **Reconstructive surgery** | Proposed surgery to approach pain issues and unpleasant feelings in the upper limb |
|  | **Medication** | Drugs prescriptions as a solution to the cancer treatment’s side effects. |
| **Physiotherapy program** | **Benefit** | Noticeable improvements and rewards for the individual. |
|  | **Difficulty** | Intrinsic difficulties or barriers related to performing the exercises. |
|  | **Knowledge** | Acquired knowledge during the physiotherapy treatment, and especially related to the reconceptualization of  pain. |
|  | **Complementary resources** | Informative brochures, therapeutic books, recording exercises or external help to do the exercises helped them learn and retain  knowledge. |
| **Physiotherapy program integration** | **Routine** | Acquire the habit of doing the daily exercises. |
|  | **Responsibility** | Moral obligation to follow the  recommendations for the success of the therapeutic process. |

|  | **Changes in activities** | Tips and resources that the participants develop from the therapeutic program. |
| --- | --- | --- |
| **Return to normal life** | **Physical exercise** | General physical activity apart from the exercises recommended by the  physiotherapist. |
|  | **Functionality** | Quality that ensures the optimal achievement of a task. |
|  | **Work** | Work activity in which movements are made or positions are maintained that may not be beneficial but cannot be modified. |
|  | **Family support** | Family support that provides help with her tasks. |
|  | **Prioritization of daily activities** | Classify the activities by order of importance based on the capacity to do them. |
